# Transition from Transbronchial Forceps to Cryobiopsy After Lung Transplantation: A Single-Centre Experience †

**DOI:** 10.3390/life14111474

**Published:** 2024-11-13

**Authors:** Davide Tosi, Margherita Brivio, Sara Franzi, Alessandro Palleschi, Gianluca Bonitta, Gianluca Lopez, Ilaria Righi, Paolo Mendogni, Margherita Cattaneo, Francesco Damarco, Letizia Morlacchi, Valeria Rossetti, Lorenzo Rosso

**Affiliations:** 1 Thoracic Surgery and Lung Transplant Unit, Fondazione IRCCS Ca’ Granda Ospedale Maggiore Policlinico, Via Francesco Sforza 35, 20122 Milan, Italy; davide.tosi@policlinico.mi.it (D.T.); margherita.brivio@unimi.it (M.B.); or alessandro.palleschi@unimi.it (A.P.); ilaria.righi@policlinico.mi.it (I.R.); paolo.mendogni@policlinico.mi.it (P.M.); margherita.cattaneo@policlinico.mi.it (M.C.); francesco.damarco@policlinico.mi.it (F.D.); or lorenzo.rosso@unimi.it (L.R.); 2 Department of Pathophysiology and Transplantation, University of Milan, Via Francesco Sforza 12, 20122 Milan, Italy; bbonit@icloud.com (G.B.); letizia.morlacchi@unimi.it; 3 Division of Pathology, Fondazione IRCCS Ca’ Granda Ospedale Maggiore Policlinico, Via Francesco Sforza 35, 20122 Milan, Italy; gianluca.lopez@policlinico.mi.it; 4 Respiratory Unit and Cystic Fibrosis Adult Center, Fondazione IRCCS Ca’ Granda Ospedale Maggiore Policlinico, Via Francesco Sforza 35, 20122 Milan, Italy; valeria.rossetti@policlinico.mi.it

**Keywords:** lung transplantation, acute cellular rejection, chronic rejection, endoscopic surveillance, transbronchial biopsy, cryobiopsy

## Abstract

The gold standard for histological acute cellular rejection diagnosis is transbronchial forceps biopsy (FB), but in recent years, transbronchial cryobiopsy (CB) has been increasingly used. This study aims to compare the diagnostic rate and safety of FBs and CBs performed in two different periods. We retrospectively reviewed our case history for the two biopsy procedures: 251 FBs (223 for surveillance purposes and 28 for clinical indication) and 218 consecutive CBs (159 for surveillance purposes and 59 for clinical indication). All biopsies were scored according to the ISHLT criteria. Diagnostic yield was higher in the CB group for all the parameters considered: a grade of acute rejection (AR) was detected in 95.0% vs. 84.5% in the CB vs. FB groups (*p* < 0.001). The diagnostic rate of airway inflammation was 65.1% vs. 51.8% (*p* = 0.005), and 89.0% vs. 64.9% (*p* < 0.001) for chronic rejection. Pneumothorax requiring chest drainage occurred in 4% of the CB group and 3% of the FB group. Moderate and severe bleeding complicated CB and FB procedures in seven (3%) and three cases (1%), respectively. Transbronchial cryobiopsies improved the diagnostic yield in the monitoring of the lung allograft. The complication rate did not increase significantly in CBs vs. FBs.

## 1. Introduction

Lung transplantation (LTx) is considered a life-saving treatment for selected patients with end-stage pulmonary disease. According to the Thoracic Transplant Registry Report of the International Society for Heart and Lung Transplantation (ISHLT), the main indications for lung transplantation are chronic obstructive pulmonary disease (COPD), interstitial lung disease (ILD), and cystic fibrosis (CF) [1].

Pulmonary complications such as acute rejection (AR) and chronic lung allograft dysfunction (CLAD) can increase morbidity and reduce post-transplant survival. AR is the most important risk factor for the development of chronic rejection and bronchiolitis obliterans syndrome (BOS). It is essential to promptly identify the early stages of AR to prevent the occurrence of sequelae or modify therapy to avoid the increased risk of CLAD development [2].

Endoscopic surveillance with transbronchial biopsy in LTx aims to lead to an early diagnosis of AR, improving long-term survival. Although new methods are being explored to detect AR at an early stage, e.g., new markers in bronchoalveolar lavage (BAL) [3,4,5,6,7,8], histopathological confirmation is still mandatory.

The gold standard for diagnosing AR involves the histological analysis of tissue fragments from a transbronchial forceps biopsy (FB). FB requires the collection of at least five samples [9] and is often associated with diagnostic errors due to small sample size, crush artefacts, and interpersonal variability in histological analysis [10]. Surgical lung biopsy would not be acceptable, because of the increased risk of complications associated with immunosuppressive therapies, such as infections or impaired wound healing [11].

Transbronchial cryobiopsy (CB) has emerged as a promising alternative. It has been used since the 1970s to diagnose various lung diseases such as interstitial lung disease (ILD), infections, and tumours [12]. Despite the scarce literature, CB has several advantages over FB: better tissue preservation, no crush artefacts, and higher yield with more alveoli and small airways [13,14]. 

Transbronchial biopsies, during post-transplant follow-up, make it possible to diagnose AR, possible airway inflammation, or chronic rejection in recipients. According to the ISHLT guidelines, graft rejection is categorized as follows:

- Acute rejection, characterized by interstitial and perivascular mononuclear infiltrates; it is classified into Grade A0 (none), Grade A1 (minimal), Grade A2 (mild), Grade A3 (moderate), and Grade A4 (severe);
- Small airway inflammation and lymphocytic bronchiolitis, classified into Grade B0 (none), Grade B1 (low grade), Grade B2 (high grade), and Grade BX (ungradeable);
- Chronic rejection, bronchiolitis obliterans; classified as Absent (C0) or Present (C1) [9].

As previously stated, few studies have compared FB and CB on diagnostic accuracy and safety [11,15], so in the present study, we reported our clinical experience in transitioning from FB to CB after lung transplantation, and, secondarily, we assessed the diagnostic yield and safety of both procedures. 

## 2. Material and Methods 

### 2.1. Patients

This is a retrospective, observational, single-centre cohort study, comparing the historical cohort of 110 lung recipients undergoing FB from January 2013 to December 2017 and the cohort of 124 patients undergoing CB from January 2018 to October 2022. 

The study was approved by the Fondazione IRCCS Ca’ Granda Ethical Committee of Milan (Ref. n 181, 24 January 2017).

In our centre, the lung transplantation protocol involves the following:

- A standard triple-drug immunosuppression therapy with prednisone, a calcineurin inhibitor (tacrolimus as first choice, or cyclosporine A when tacrolimus-due neurotoxicity is documented), and a cytostatic agent (azathioprine or mycophenolate mofetil) [16];
- A routine follow-up with transbronchial biopsy (TBB) at three, six, and twelve months from surgery, defined as surveillance biopsies (SBs).

Before the TBB procedure, a sample of BAL is obtained, in the middle lobe or lingula or in the affected lobe according to radiological findings, to evaluate the presence of infections. Biopsies are also performed, outside of scheduled intervals, for clinical indications (CIBs), such as unexplained dyspnea, fever, asthenia, nonproductive cough, hypoxemia, or asymptomatic decline > 10% in forced expiratory volume in 1 s (FEV1) compared to baseline. Table 1 summarizes the demographic characteristics of the patients enrolled in the study (Table 1). Patients were stratified into three groups, according to the risk for cytomegalovirus (CMV) infection and disease: low-risk (D−/R−); intermediate-risk (R+); and high-risk (D+/R−) (D: donor; R: recipient) [17].

### 2.2. Biopsy Technique 

All the collected procedures were performed in the operating theatre under conscious sedation, using hypnotic drugs (Midazolam or Propofol) plus an analgesic drug (Remifentanil or Ketamine). Local anesthesia was performed, with 2% Lidocaine to the oropharynx at the beginning of the procedure.

Spontaneous breathing was maintained during the entire procedure. Oxygen was insufflated constantly through a nasal cannula. Blood pressure, oxygen saturation, electrocardiogram, and transcutaneous carbon dioxide partial pressure were continuously monitored. 

The flexible Olympus (Olympus, Tokyo, Japan) bronchoscope was introduced nasally or orally into the selected bronchus. In SBs, in the absence of clinical and radiological contraindications, the biopsy is performed in the right lower or middle pulmonary lobe, to place a bronchial blocker in case of major bleeding.

The cryoprobe (Erbe 2.4 mm, Elektromedizin GmbH, Tübingen, Germany) was introduced through the working channel of the bronchoscope. Once in position, the probe was cooled with CO2 for approximately four seconds, until the probe’s tip temperature reached −89 °C. After that, the entire bronchoscope was retracted, with the frozen lung tissue attached to the probe’s tip. The frozen sample was fixed in formalin and sent for histopathological assessment. The collection of two well-ventilated, macroscopically adequate parenchyma samples was considered sufficient.

After each biopsy, the bronchoscope was re-introduced to check for bleeding and determine the extent of it. Bleeding was controlled by suctioning with a flexible bronchoscope; in case of significant bleeding, further procedures were undertaken, such as cold topical saline (up to a maximum of 80 mL), tranexamic acid (up to 1 g), or adrenaline instillation (up to 0.2 mg). 

One hour after the procedure, a chest X-ray was taken at the bedside, in a semi-sitting position, to exclude pneumothorax.

### 2.3. Histological Analysis and Therapeutic Approaches

The biopsy samples were scored for acute rejection (A0–A4), airway inflammation (B0–B2 and Bx), and chronic rejection (C0–C1), following the ISHLT criteria [9]. Based on these histological results, different therapeutic approaches are established: patients with AR grade 2 or higher might follow a standard treatment even without clinical symptoms. Patients with AR grade 1 and the presence of symptoms can also be treated. Standard treatment involves a 3-day pulse with high doses of intravenous corticosteroids (methylprednisone 10 mg/kg/day), followed by slow tapering. In our centre, patients with AR grade 1 without clinical signs start intravenous corticosteroids (0.5–1 mg/kg/day).

### 2.4. Statistical Analysis

Continuous data were presented as mean and standard deviation. Binary data were shown as absolute and percentage frequencies and were compared by performing the *t*-test for independent data or a Z-test, as appropriate.

Diagnostic yield, bleeding, and pneumothorax were analyzed using generalized estimating equation (GEE) regression, computed on total procedure number [18]. The GEE model was performed using a log for the link function to compute Relative Risk (RR)—link to identify per risk difference, the sandwich estimator for standard error, and the unstructured working correlation matrix selected by correlation information [19]. The GEE regression model was adjusted by the variables sex, primary graft dysfunction (PGD), surveillance CIB, EVLP, and CMV infection grade into a linear predictor, without any interaction terms. The GEE regression allows for a population-averaged interpretation of the regression coefficients.

A univariate Wald test for each GEE-estimated parameter was performed. Wald confidence intervals (Cis) were also computed at 95%. All statistical tests were 2-sided and a *p*-value < 0.05 was considered statistically significant.

The inference should be considered exploratory. All the analyses were carried out using the R software (version 4.3.0) [20,21,22].

## 3. Results

From January 2013 to December 2017, we collected 251 FBs (from 110 patients), of which 223 were for surveillance and 28 for clinical indication. Between January 2018 and October 2022, we collected 218 CBs (from 124 patients), of which 159 were for surveillance and 59 for clinical indication. 

The grade of AR was detected in 95.0% of cases in the CB group vs. 84.5% in the FB group (*p* < 0.001). The diagnostic rates of airway inflammation were 65.1% and 51.8% (*p* = 0.005), respectively. Chronic rejection was diagnosed in 89.0% vs. 64.9% in CBs vs. FBs (*p* < 0.001). As we previously found, among FBs, the CIB diagnosis rate was 96%, with only one procedure found to be non-diagnostic. The diagnostic rate of acute rejection with CIBs was significantly higher than with SBs (36% vs. 4%, *p* > 0.0001) [16]. Our recent findings concerning CBs show no differences in the diagnostic rate of CIBs and SBs (95%) (Table 2).

Regarding adverse events, the complication rate was similar in the two techniques. In the FB group, seven patients (3%) had pneumothorax requiring chest drainage and three patients (1%) had moderate bleeding [16]. In the CB group, eight patients (4%) had pneumothorax requiring chest drainage and seven patients (3%) had moderate bleeding, treated with adrenaline or tranexamic acid (Table 3).

No patients undergoing FB or CB required blood transfusions, and no deaths occurred.

The non-adjusted GEE analysis showed that CB has a significantly lower risk of being a non-diagnostic procedure than FB (RR 0.58; 95% CI 0.48–0.71; *p* < 0.001). Likewise, the adjusted GEE analysis showed that CB has a significantly lower risk of being a non-diagnostic procedure, compared to FB (RR = 0.663; 95% CI 0.53–0.83; *p* < 0.001) (Table 4).

## 4. Discussion

In our retrospective study, we compared the two procedures, CB and FB, performed in lung transplant patients, during two time periods: until 2017, the standard technique was FB, but since January 2018, we have been using CB. Cryobiopsies demonstrated a higher diagnostic yield compared to FBs, with no increase in the rate of complications. This finding highlights the potential of CB to improve diagnostic accuracy in the post-Tx setting while maintaining a similar safety profile to traditional FB. In our experience, CB provided samples of superior quality, with larger tissue size and fewer crush artefacts, thereby enhancing the likelihood of accurately diagnosing AR with fewer biopsy specimens. This advantage reduces the duration of the procedure, also minimizing patient distress.

The benefits of CB have previously been demonstrated in patients with parenchymal lung disease and lung cancer [12,23]. However, few and no recent data have been published on the diagnostic efficacy of transbronchial CBs in lung transplants [24,25]. Nowadays, the gold standard for the diagnosis of AR is FB, but, as already described by Pourabdollah, small specimens and crushed artefacts often make diagnosis difficult [10]. In our experience, CB seems to be a promising alternative procedure [2], and indeed CB yields larger biopsies with preserved histology and free of artefacts, with a higher number of alveoli and small airways [13].

In our case history, the diagnostic rate of CB on FB is quite similar looking at all parameters. However, regarding airway infections and chronic rejection, CB was more diagnostic than FB (Table 2). Moreover, FB showed a different yield in the case of clinically indicated or surveillance biopsies being more diagnostic in CIB for acute rejection (36% vs. 4%). On the contrary, cryobiopsy did not show any statistical differences between CIBs and SBs, with a diagnostic rate of 95% over FB for acute rejection. This renders CBs more appealing since it makes it possible to identify the early stages of AR in a larger number of non-symptomatic patients, thus facilitating the prompt initiation of therapy and the prevention of chronic rejection. Previously, we reported a higher diagnostic rate of CB over FB in AR detection (100% vs. 83%), as well as for airway inflammation and chronic rejection [2]. The adjusted GEE analysis in Table 4 shows that CB has a relatively lower risk of being non-diagnostic (RR = 0.663) than FB. 

Our findings are in line with what is published in the literature: Gersham in 2018 reported a significantly higher diagnostic rate of CB over FB in AR detection (21% vs. 14.9%) [15]. More recently, Steinack compared the diagnostic power of FB and CB for AR and found similar results (28.6% vs. 4.8%) [11].

Regarding complications, we observed similar complication rates between the two procedures in our study: as shown in Table 3, the slightly higher rates of pneumothorax or moderate bleeding in CB over FB groups (eight vs. seven and seven vs. three, respectively) are not significantly different, similarly to what was previously reported by our team [16]. Loor et al. reported on such complications, as pneumothorax and bleeding [26,27]. Indeed, both are the most frequent adverse events during CBs, yet there is no evidence of a higher incidence in CBs than in FBs. However, the authors recommend careful monitoring of blood pressure to prevent bleeding. The adjusted GEE analysis showed that CB and FB have comparable bleeding rates and pneumothorax occurrence (RR= 0.99; 95% CI 0.98–1.01, *p* = 0.8502 and RR 1.9, 95% CI 0.50–3.85, *p* = 0.5373, respectively). The similar complication rate may be related to the fewer samples performed in the CB group; indeed, once two samples of well-ventilated parenchymal tissue had been obtained, the procedure ended.

Our findings are in line with what has already been published. Previous studies have come to similar conclusions: in 2018, Gershman reported 4.5% cases of pneumothorax in the CB group and 4% in the FB group. The bleeding rate was also similar; all bleeding cases were treated with topical cold water or hexakapron, without the need for blood transfusion or surgical intervention [15]. Roden observed more complications in CBs than in FBs; nevertheless, the differences were not significant [13]. Apart from the evaluation of adverse events, Awano et al. in 2024 reported a similar safety profile between cryobiopsies and forceps biopsies [28].

Our study has several limitations: it is a retrospective study based on medical and pathological records, and it is a single-centre study; moreover, the relatively small sample size limits the generalizability of the results.

## 5. Conclusions

In conclusion, considering the higher diagnostic yield of the CBs for all the considered histological parameters and the similar complication rates, cryobiopsies were demonstrated to be safe and effective for diagnosing lung AR, compared with conventional forceps biopsies. These results emphasize their potential and will probably lead to an increasing application of this technique in LTx. Although the small number of cases included and the retrospective nature of our study do not allow us to generalize the results obtained, our findings are concordant with the literature and highlight the good diagnostic adequacy of CBs. Certainly, larger prospective studies are needed to confirm these results.

## Figures and Tables

**Table 1:** Demographic characteristics of the patients enrolled in the study.

Patients’ Characteristics		FB (110 Patients)	CB (124 Patients)	*p*-Value
Sex, male, n (%)		57 (51.8)	73 (58.9)	0.3412
Age, years, mean (SD)		44 (14.0)	40 (14.0)	0.0301
Underlying disease	Cystic fibrosis, n (%)	52 (47.3)	68 (54.8)	0.3055
	IPF, n (%)	31 (28.2)	22 (17.7)	0.0805
	COPD, n (%)	8 (7.3)	13 (10.5)	0.5296
	Miscellanea, n (%)	19 (17.3)	21 (16.9)	ns
Bilateral transplantation, n (%)		86 (78.2)	121 (97.6)	<0.001
CMV risk classes	CMV low risk, n (%)	26 (23.6)	37 (29.8)	0.3576
	CMV intermediate risk, n (%)	61 (55.5)	81 (65.3)	0.1590
	CMV high risk, n (%)	23 (20.9)	6 (4.8)	0.0004
EVLP, n (%)		8 (7.3)	29 (23.4)	0.0014
Grade 3 primary graft dysfunction, n (%)		39 (35.5)	14 (11.3)	<0.001

Abbreviations. FB: forceps biopsy; CB: cryobiopsy; SD: standard deviation; IPF: idiopathic pulmonary fibrosis; COPD: chronic obstructive pulmonary disease; CMV: cytomegalovirus; EVLP: ex vivo lung perfusion.

**Table 2:** Details of the histopathological results according to the ISHLT grading scale.

Parameters		CB		FB		*p*-Value
		SBs	CIBs	SBs	CIBs	
Procedures, n (%)		159 (73)	59 (27)	223 (89)	28 (11)	
Diagnostic yield	AR, n (%)	207 (94.95)		212 (84.5)		*p* < 0.001
	AI, n (%)	142 (65.13)		130 (51.8)		*p* < 0.001
	CR, n (%)	194 (88.9)		163 (64.9)		ns
	All parameters, n (%)	103 (64.7)	37 (62.7)	90 (40.4)	12 (42.8)	

Abbreviations. ISHLT: International Society Heart and Lung Transplantation; CB: cryobiopsy; FB: forceps biopsy; SB: surveillance biopsy; CIB: clinically indicated biopsy; AR: acute rejection; AI: airway inflammation; CR: chronic rejection.

**Table 3:** Complication rates of FB and CB procedures.

Complications	CB (n = 218)	FB (n = 251)	*p*-Value
Bleeding, n (%)	7 (3.2)	3 (1.9)	0.2353
PNX (with CD), n (%)	8 (3.7)	7 (2.7)	0.7813

Abbreviations. CB: cryobiopsy; FB: forceps biopsy; PNX: pneumothorax; CD: chest drainage.

**Table 4:** Adjusted GEE regression.

GEE Regression Variables	Point Estimation (RR)	95% CI	*p*-Value
Intercept	0.460	0.35–0.61	<0.001
Forceps/Cryo = 1	0.663	0.53–0.83	<0.001
PGD	1.198	1.04–1.39	0.0166
Surveillance CIB	1.01	0.87–1.21	0.8304
EVLP	1.03	0.91–1.63	0.6035
CMV medium risk	1.27	0.97–1.67	0.0856
CMV high risk	1.41	1.05–1.90	0.0241
Bilateral vs. Unilateral	1.38	1.18–1.60	<0.001

Abbreviations. GEE: generalized estimating equation; RR: relative risk; CI: confidence interval; PGD: primary graft disease; CIB: clinically indicated biopsy; EVLP: ex vivo lung perfusion; CMV: cytomegalovirus.

## Data Availability

The article’s data will be shared on reasonable request to the corresponding author.
